# Green synthesis, characterization and biological activities of silver nanoparticles synthesized from *Neolamarkia cadamba*

**DOI:** 10.5599/admet.1793

**Published:** 2023-07-01

**Authors:** Juluri Maheswari, Mohammed Reshma Anjum, Mohan Sankari, Golla Narasimha, Suresh Babu Naidu Krishna, Battini Kishori

**Affiliations:** 1 Department of Biotechnology, Sri Padmavati Mahila Visvavidyalayam (Women’s University), Tirupati- 517 502, A.P. India; 2 Department of Virology, Sri Venkateswara University, Tirupati- 517 502, A.P. India; 3 Institute for Water and Wastewater Technology, Durban University of Technology, Durban – 4000, South Africa

**Keywords:** Antibacterial activity, antifungal activity, antioxidant activity, electron microscopy

## Abstract

**Background and purpose:**

Metal nanoparticles are essential due to their unique catalytic, electrical, magnetic, and optical characteristics, as well as their prospective use in sensing, catalysis, and biological research. In recent years, researchers have focused on developing cost-effective and eco-friendly biogenic practices using the green synthesis of metal nanoparticles (AgNP).

**Experimental approach:**

In the present study, the aqueous extracts prepared from the leaf, stem, bark, and flower of *Neolamarkia cadamba* were used for the synthesis of silver nanoparticles. Synthesized silver nanoparticles were characterized using UV-Visible spectroscopy, zeta potential, dynamic light scattering, scanning electron microscope (SEM), and EDAX.

**Key results:**

The current study showed absorption of synthesized AgNPs at 425, 423, 410, and 400 nm. Dynamic light scattering of AgNPs Showed size distribution of AgNPs synthesized from leaf, stem, and flower aqueous extracts ranges from 80-200 nm and AgNPs prepared from bark extract ranges from 100-700 nm. Zeta-potential of the biosynthesized AgNPs was found as a sharp peak at -23.1 mV for the leaf, -27.0 mV for the stem, -34.1 mV for the bark, and -20.2 mV for the flower. Silver nanoparticles and crude extracts of *Neolamarkia cadamba* showed effective antibacterial, antifungal, and antioxidant activities.

**Conclusion:**

Silver nanoparticles have substantial antibacterial activity against Gram-positive bacteria and also exhibit the utmost antifungal activity against *Aspergillus niger*. The study concludes that the green synthesis of silver nanoparticles from *N. cadamba* leaf, stem, bark, and flower extract is a reliable and eco-friendly technique.

## Introduction

Metal nanoparticle synthesis has emerged in material science in recent years due to its applications in medicine, agriculture, drug delivery, environmental bioremediation, and information storage [1-5] due to its unique optical, catalytic, magnetic, electronic, and antimicrobial properties [6-10]. Silver is preferred for the synthesis of nanoparticles due to its antibacterial and catalytic characteristics and is non-toxic to humans in comparison with other metals [11]. Nanocrystalline silver particles are often used in high-sensitivity biomolecular detection, therapeutics, catalysis, antimicrobials, and microelectronics [12-15]. Silver nanoparticles (AgNPs) can be synthesized by physical, chemical, and biological methods. Physical as well as chemical methods are exorbitant, time-consuming, and non-eco-friendly. They require a large quantity of energy, toxic solvents, and hazardous chemicals [16]. The biological method is inexpensive, energy-efficient, non-toxic, and eco-friendly [17]. Fungi, bacteria, yeast, and plant sources are used in the synthesis of AgNPs, which is termed green synthesis [18,19]. Green synthesis utilizing plants is common; it is safe, economical, and harmless to the environment [20].

*Neolamarkia cadamba* is a member of the *Ruiaceae* family. This plant is commonly referred to as 'Kadam'. Many Indian groups utilize its bark, leaf, flower, stem, and root to treat a variety of diseases, such as fever, sore throat, cough, inflammation, and infections [21,22]. The pharmacological properties of *Neolamarkia cadamba* involve anticancer [23], antiprotozoal [24], antidiabetic [25], antibacterial [26], antifungal [27], antioxidant [28], anti-inflammatory [29], wound healing [30] and anti-malarial activity [22].

In the present study, AgNPs were synthesized by eco-friendly green synthesis using aqueous leaf, stem, bark, and flower extracts of *Neolamarkia cadamba* at ambient temperature. Characterization of biosynthesized AgNPs was done by using UV-visible spectroscopy, zeta-potential, dynamic light scattering (DLS), scanning electron microscopy (SEM), and energy dispersive X-ray analysis (EDAX). Antibacterial activity was tested on selective bacterial strains like *Staphylococcus aureus* and *Bacillus subtilis, Gram-positive, and Pseudomonas aeruginosa and Escherichia coli*, Gram-negative bacteria. The DPPH method was used to determine antioxidant and antifungal activity against *Aspergillus niger*.

## Experimental

### Collection of samples

**Figure 1.**
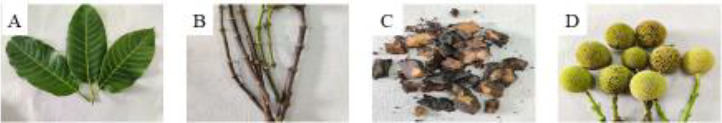
*N. cadamba* (A) leaf, (B) stem, (C) bark and (D) flower

*Neolamarkia cadamba* leaves, stems, bark, and flowers were collected in Tirupati, Chittoor district, Andhra Pradesh, India. The fresh plant parts were gathered in polyethylene zipper bags to eliminate filth, cleaned with tap water, and then distilled water. After that, the samples were shadow dried at room temperature. The samples were then crushed with an electric mixer and put in airtight polyethylene bottles until they could be looked at more closely (Figure 1).

### Chemicals

All the chemicals used were analytical grade.

### Plant extract preparation

Aqueous extracts of the leaves, stem, bark, and flowers were prepared using distilled water (150 ml) in four different Erlenmeyer flasks of 500 ml each and positive control in a separate flask, followed by heating at 70–80 °C in a water bath for 2-3 hours and later cooling at room temperature. The obtained extracts were centrifuged for 5 minutes at 3000 rpm. The recovered supernatant was filtered through Whatman No. 1 filter paper using a Buchner funnel. The filtrate was stored at 40 °C and used for further analysis.

### Silver nitrate stock solution

To prepare 1 mM silver nitrate solution, 17 mg of silver nitrate was dissolved in 100 ml of distilled water and stored in an amber glass bottle.

### Characterization of silver nanoparticles

Ten milliliters of AgNO_3_ (1 mM) solution was added to 1 ml of plant extract and made up to 15 ml using distilled water. The color shift of the solution from colorless to brown verified the reduction of Ag+ to Ag^0^. To eliminate errors caused by the high optical density of the solution, distilled water was added ten times to the particle suspension.

### UV-Visible analysis

The bio-reduction of pure Ag^+^ ions was evaluated by sampling aliquots (0.5 ml) and diluting the samples with 5 ml of de-mineralized water, and the samples were further analyzed using UV–Vis spectroscopy (Perkin-Elmer Lambda 25 spectrophotometer) [31]. Absorption peak in the range of 350 to 500 nm revealed the existence and reduction of silver ions.

### Dynamic light scattering (DLS)

DLS (Malvern Instruments Ltd., UK) was used to assess particle size distributions by monitoring dynamic variations in light scattering intensity caused by the Brownian motion of the particles. The AgNPs were diluted and either filtered through a 0.22 μm syringe-driven filter or left unfiltered [17].

### Zeta potential measurements

The physical attribute that determines the net surface charge of nanoparticles was defined as zeta potential. When the zeta potential values ranged from higher than +30 mV to less than -30 mV, the requirements for nanoparticle stability were evaluated [32]. The pH of the samples was then adjusted to the desired amount and vortexed for 30 minutes. After the vortex, the zeta potential was measured and the equilibrium pH was determined. An average of three distinct measures was reported in each case [33].

### Scanning electron microscopy (SEM) analysis

Scanning electron microscopy characterizes the morphology and size of synthesized AgNPs. A mercury lamp was used to dry the films on the carbon-coated copper grid for 5 minutes. SEM micrographs were taken on a ZEISS EVO 40 Electron microscope [34].

### EDAX measurements

EDAX analysis was performed using a HITACHI SU6600 FE-SEM fitted with EDAX attachment after drying the AgNPs on a carbon-coated copper grid at 25 °C. The X-rays were detected using a semiconductor material combined with circuits to analyze the spectrum [35].

### Antibacterial activity

The crude extracts and AgNPs synthesized from aqueous extracts of *N. cadamba* were tested for antibacterial activity using the agar well diffusion technique against Gram-positive bacteria *S. aureus* and *B. subtilis*, as well as Gram-negative bacteria *E. coli* and *P. aeruginosa*. To do this, 100 μl of active culture inoculums were added to the nutrient agar plates in a clean way and spread out over the medium. A sterile borer was used to create 5 wells of 8 mm diameter on 4 mm thick nutrient agar plates. Various concentrations of crude and AgNPs samples (20, 40, 60, and 80 μg/μl) were placed in four wells, with streptomycin (Himedia) (10 μg/μl) serving as a positive control in one well. Incubation was done on these agar plates at 37 °C for 24 to 48 hours. The experiment was performed in duplicate to minimize errors. After incubation, each plate was examined, and zones of inhibition were measured using a ruler [36,37].

### Antifungal activity

The crude extracts and AgNPs synthesized from aqueous extracts of various plant components, *i.e.*, leaf, stem, flower, and bark, were investigated using the agar well diffusion method for antifungal activity. *Aspergillus niger* (ATCC 9029) was obtained from the Microbial Type Culture Collection in Chandigarh, India. *A. niger* stock cultures were developed and placed at 4 °C. Plates of potato dextrose agar was prepared and solidified. To inoculate 100 μl (0.1 ml) of fungal culture, the spread plate method was used. Borers were used to drill wells, which were then filled with varying concentrations of crude extracts and synthesized silver nanoparticles, with one well serving as a control. These plates were incubated for 7 days at 26 ± 4 °C. After incubation, a clear zone was formed around the well, which was examined and measured with a ruler. Antifungal activity was indicated by the formation of a clear zone [38,39].

### Antioxidant assay (DPPH method)

With minor modifications, the procedure was conducted by following the protocol of Bhakya *et al*. [40]. Using the stable radical DPPH, the scavenging activity of free radicals from crude extracts and AgNPs, along with standard vitamin C, were measured. 1 ml of crude extracts and AgNPs at various concentrations (40, 80, 120, 160, 200, and 400 μg/μl) were mixed with freshly prepared 1 ml of DPPH solution (0.1 mM in methanol) and vortexed vigorously. The solution was then incubated in the dark for 30 minutes at room temperature. The absorbance was measured at 517 nm using a UV-Vis spectrophotometer. DPPH (all reagents except the sample) was employed as a control, while methanol served as a blank solution. The scavenging activity of free radicals of AgNPs generated from different portions of plant extracts was represented as a percentage of inhibition, calculated using equation (1).


(1)

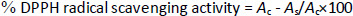



where *A*_c_ is the control absorbance of DPPH radical + methanol and *A*_s_ is the sample absorbance of DPPH radical + sample AgNPs / vitamin C.

## Results

### Color change and UV-vis spectroscopy

**Figure 2.**
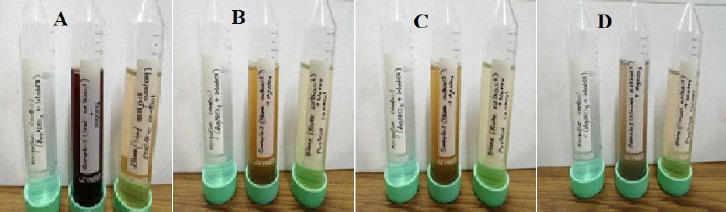
Colour change indicates the formation of AgNPs synthesized from aqueous extracts of: (A) leaf, (B) stem, (C) bark and (D) flower *of N. cadamba*

Nanoparticles began to form after the extract was mixed with silver nitrate solution. The formation of AgNPs was confirmed by the observable changes in the solution's color, as shown in Figure. 2, *i.e.*, from pale yellow to brown.

**Figure 3.**
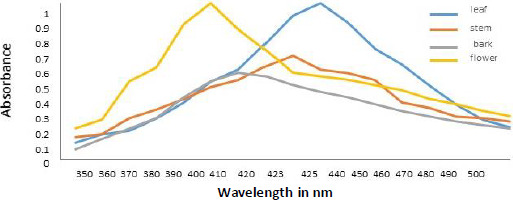
UV–Vis absorption spectra of AgNPs synthesized from aqueous extracts of leaf, stem, bark and flower of *N.* cadamba

Spectral analysis was another method of verifying developed AgNPs. The absorption of synthesized AgNPs from leaf, stem, bark, and flower aqueous extracts of *N. cadamba* (Figure 3) was observed at 425, 423, 410, and 400 nm due to surface plasmon resonance (SPR).

### Dynamic light scattering of silver nanoparticles

**Figure 4.**
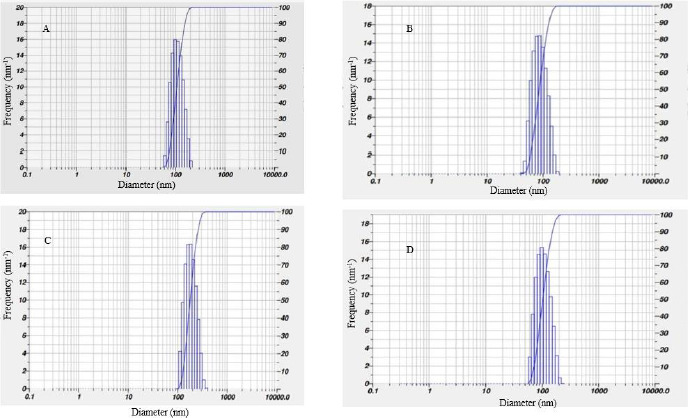
DLS showing average size of AgNPs synthesized from extracts of (A) leaf, (B) stem (C) bark (D) flower of *N. cadamba*

Current observation shows that the size distribution of AgNPs synthesized from leaf, stem and flower aqueous extracts ranges from 80–200 nm and that AgNPs prepared from bark extract range from 100 to 700 nm. The calculated Z-average particle size distribution of synthesized AgNPs from leaf extract was 77.5 nm, stem extract was 80.9 nm, bark extract was 757.7 nm, and flower extract was 11559.6 nm, as shown in below Figure 4.

### Zeta potential of silver nanoparticles

**Figure 5.**
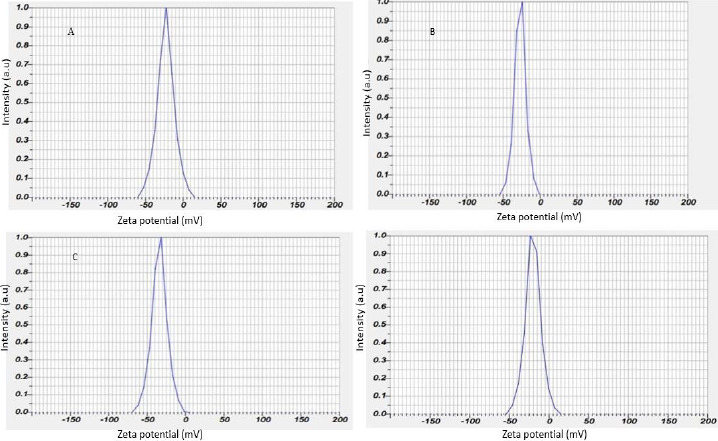
Zeta potential of AgNPs synthesized from extracts of (A) leaf (B) stem (C) bark and (D) flower of *N. cadamba*

The zeta potential of the biosynthesized AgNPs was found as sharp peak at -23.1 mV for the leaf, -27.0 mV for the stem, -34.1 mV for the bark and -20.2 mV for the flower, as shown in Figure 5.

### Scanning electron microscopy (SEM)

**Figure 6.**
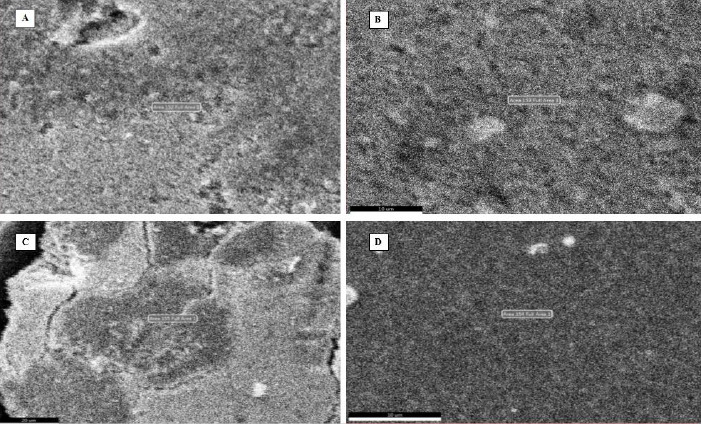
SEM micrographs of AgNPs synthesised from extracts of (A) leaf (B) stem (C) bark and (D) flower of *N. cadamba*

Figure 6 illustrates the SEM images of synthesized silver nanoparticles from leaf, stem, bark, and flower aqueous extracts of *N. cadamba.* The results of SEM concluded that AgNPs size and spherical form were not defined due to agglomeration.

### EDX of silver nanoparticles

**Figure 7.**
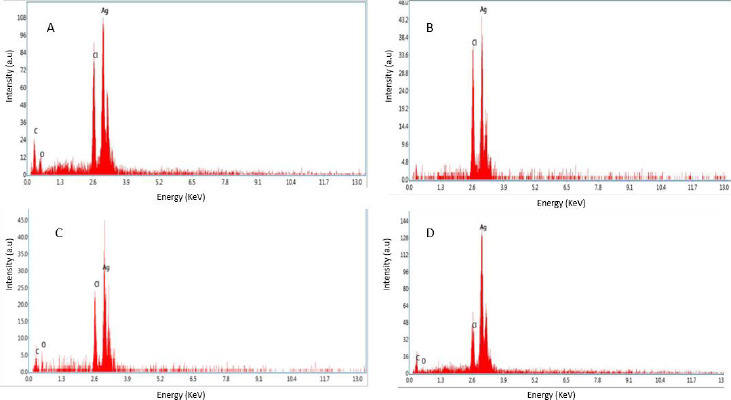
EDAX spectra of AgNPs synthesized from extracts of (A) leaf (B) stem (C) bark (D) flower of *N. cadamba*

The observations from energy dispersive spectroscopy show very high silver signals and weak chloride and carbon signals, indicating that the conversion of silver ions to silver elements may come from molecules linked on the surface of AgNPs. Silver nitrate was reduced to silver nanoparticles, as evidenced by the dense peak of silver. The silver peak is significantly thicker than the others in the spectrum, as shown in Figure 7.

### Antibacterial activity

**Figure 8.**
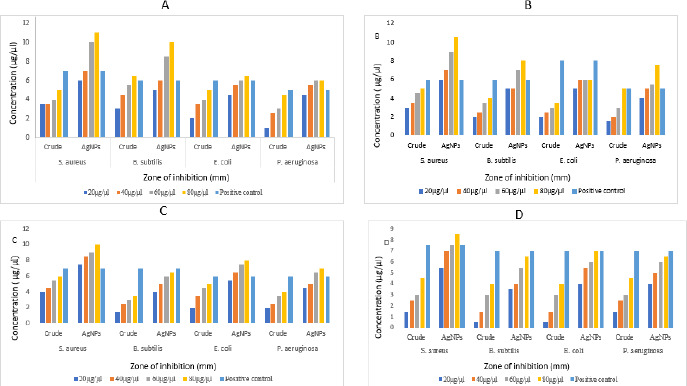
Antibacterial activity of AgNPs synthesized from extracts of (A) leaf (B) stem (C) bark and (D) flower of *N. cadamba*

The aqueous crude and AgNPs extracts from leaf, stem, bark and flower of *N. cadamba* have potential biological activity towards antibacterial on four different bacterial strains like *B. subtilis, S. aureus, E. coli* and *P. aeruginosa*. Both crude and AgNPs of *N. cadamba* showed different zone of inhibitions at concentrations of 20, 40, 60 and 80 μg/μl along with streptomycin as a positive control, as shown in Figure 8.

### Antifungal activity

**Figure 9.**
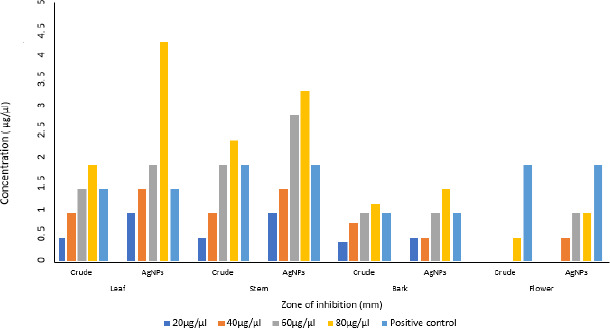
Antifungal activity of AgNPs synthesized from (A) leaf (B) stem (C) bark and (D) flower of *N. cadamba*

Aqueous crude and AgNPs extracts of leaf, stem, bark and flower of *N. cadamba* showed antifungal activity against *A. niger* (Figure 9*)* compared to crude extracts.

### Antioxidant activity

**Figure 10.**
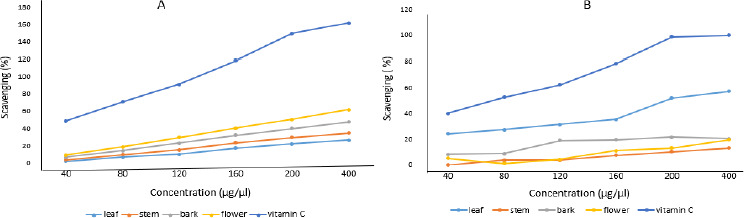
Antioxidant activity of (A) *N. cadamba* and (B) crude extracts.

Aqueous crude and silver nanoparticle extracts of leaf, stem, bark and flower of *N. cadamba* tested positive for antioxidant properties. It is confirmed that both crude and silver nanoparticle extracts synthesized from different parts of *N. cadamba* show antioxidant activity depending on the relative dosage. AgNPs prepared from different parts of *N. cadamba* showed effective antioxidant activity, as shown in Figure 10 (A), compared to crude extracts, as shown in Figure 10 (B).

## Discussion

The present work describes the green synthesis of AgNPs with the help of aqueous extracts of the leaf, stem, bark, and flower of *N. cadamba*. Green synthesis of AgNPs was popular due to the absence of harmful ingredients, low cost, eco-friendly, and suitable for biomedical and pharmaceutical applications [41]. The color of the silver nitrate solution was changed from pale yellow to brown after the aqueous extracts were added to it. The reduction of silver nitrate into AgNPs was confirmed by the development of a brown-colored solution [42,43]. The reduction of Ag+ to Ag^0^ is directly proportional to the concentration of crude extract, similar results were observed during the synthesis of AgNPs using olive leaf extract [44].

Colloidal AgNPs were excited by absorbing light in the range of 400 to 450 nm due to surface plasmon resonance [45]. The excitation peak observed using a UV-vis spectrophotometer indicated the presence of AgNPs. Compared to the strong SPR peak obtained in the UV-vis spectra, the broad spectrum of the DLS analyzer reveals that the particle size was smaller [46]. The surface of the nanoparticles was thought to be negatively charged and was disseminated in the medium. The negative values in zeta potential confirmed the repulsion and stability among the particles [47]. The observations from energy dispersive spectroscopy show very high silver signals and weak chloride and carbon signals [48], indicating the reduction of silver ions to elemental silver, which may have come from molecules linked to the AgNPs surface. The silver peak is significantly thicker than the other peaks [49]. Recent studies revealed that most bacteria gained resistance to recurrently used antibiotics, necessitating the use of alternate medicines [50].

AgNPs showed effective antibacterial activity compared to crude extract against Gram-positive and Gram-negative bacteria, similar studies on *Cucumis prophetarum* revealed that nanoparticles showed enhanced activity against bacterial pathogens compared to crude extracts [51,52]. The studies show less zone of inhibition against *Aspergillus niger* at performed concentrations [53]. The color changes were observed upon mixing AgNPs with DPPH solution, indicating the scavenging activity of DPPH due to the donation of hydrogen atoms, which revealed that AgNPs synthesized from *N. cadamba* displayed effective antioxidant properties compared to crude, studies on AgNPs synthesized from *Elephantopus scaber* leaf extract exhibited similar results [54].

## Conclusion

The development of a sustainable and environmentally friendly approach for the synthesis of metallic nanoparticles are a key necessity in the field of nanotechnology. Nanoparticles are regarded as essential building components in nanotechnology. Because of their appealing physiochemical characteristics, silver nanoparticles play a significant role in biological research. The current study explains the development of silver nanoparticles using aqueous extracts of *N. cadamba* leaf, stem, bark, and flower. Bioactive compounds present in the leaf, stem, bark, and flower of *N. cadamba* show a color change when silver nitrate is reduced to silver nanoparticles. It proves to be an environment-friendly, quick green approach to synthesis, as well as a cost-effective and efficient way to make silver nanoparticles. Characterization was done by using methods like UV-Vis absorption spectrophotometer, DLS, Zeta-potential, SEM, and EDAX to the extracted silver nanoparticles. They prove that the capping agent provides stability to the AgNPs and also the formation of AgNPs. The phytochemical screening confirms the occurrence of tannins, steroids, cardiac glycosides, saponins, terpenoids, flavonoids, and alkaloids. The green synthesized silver nanoparticles show effective antibacterial activity towards *B. subtilis, S. aureus, E. coli*, and *P. aeruginosa*, antifungal activity towards *A. niger*, and antioxidant activities when compared with crude extracts. The AgNPs synthesized from *N. cadamba* were less effective against *A. niger* and *C. albicans* at performed concentrations.

AgNPs from leaf extract of *N. cadamba* showed effective antibacterial, antifungal, and antioxidant activities compared to AgNPs synthesized from stem, bark, and flower extracts of *N. cadamba*. The silver nanoparticles synthesized from plant extracts could be used in medicine, food, drug delivery, and in the preparation of pharmaceuticals such as antibiotics because of low cost, non-toxic, organic, and most efficacious against bacteria and fungi, which cause diseases in humans and plants. In the future, we are also planning to do the anticancer and antidiabetic activity of AgNPs synthesized from the *N. cadamba* plant.
